# Development and Analysis of Mechanical Properties of Caryota and Sisal Natural Fibers Reinforced Epoxy Hybrid Composites

**DOI:** 10.3390/polym13060864

**Published:** 2021-03-11

**Authors:** Ayyappa Atmakuri, Arvydas Palevicius, Lalitnarayan Kolli, Andrius Vilkauskas, Giedrius Janusas

**Affiliations:** 1 Faculty of Mechanical Engineering and Design, Kaunas University of Technology, Studuntu 56, 01513 Kaunas, Lithuania; arvydas.palevicius@ktu.lt (A.P.); andrius.vilkauskas@ktu.lt (A.V.); giedrius.janusas@ktu.lt (G.J.); 2 Faculty of Mechanical Engineering, Sir C.R. Reddy College of Engineering, Vatluru, 522403 Andhra Pradesh, India; klalitnarayan@gmail.com

**Keywords:** Caryota and sisal fibers, epoxy resin, hybrid composites, mechanical properties, moisture absorption, SEM analysis

## Abstract

In recent years, natural fiber reinforced polymer composites have gained much attention over synthetic fiber composites because of their many advantages such as low-cost, light in weight, non-toxic, non-abrasive, and bio-degradable properties. Many researchers have found interest in using epoxy resin for composite fabrication over other thermosetting and thermoplastic polymers due to its dimensional stability and mechanical properties. In this research work, the mechanical and moisture properties of Caryota and sisal fiber-reinforced epoxy resin hybrid composites were investigated. The main objective of these studies is to develop hybrid composites and exploit their importance over single fiber composites. The Caryota and sisal fiber reinforced epoxy resin composites were fabricated by using the hand lay-up technique. A total of five different samples (40C/0S, 25C/15S, 20C/20S, 15C/25S, 0C/40S) were developed based on the rule of hybridization. The samples were allowed for testing to evaluate their mechanical, moisture properties and the morphology was studied by using the scanning electron microscope analysis. It was observed that hybrid composites have shown improved mechanical properties over the single fiber (Individual fiber) composites. The moisture studies stated that all the composites were responded to the water absorption but single fiber composites absorbed more moisture than hybrid composites.

## 1. Introduction

A matrix material plays an important role in composite fabrication. A matrix is used as a load distributer among the reinforcement material when the external pressure is applied. Based on the matrix material polymer composites are classified as thermoset polymers and thermoplastic polymer composites [1,2]. Unlike thermoplastics, thermosets are not recyclable because it does not come back to its original state when the resin is converted from liquid state to solid state after the curing process. Just heating process is required for thermoplastics to get a new shape. Many researchers found interest in using the thermoset polymer resins over thermoplastic resin due to its wide range of applications. Among all the thermoset resins, epoxy resins have gained much popularity due to their adhesive properties, low shrinkage and curing time, good permeability resistance to moisture [3,4,5,6,7]. Due to their high adhesive properties, epoxy resins are vastly suited for bonding with various materials, such as fibers, steel, plastics, and wood [8,9]. To create a strong network-like structure between reinforcement and matrix material, a curing agent (hardener) is usually added with the epoxy resin. Though their great cross-linking density leads to the crack propagation and fracture toughness of the composites. Many researchers have shown interest to improve its structural stability by using chemical and physical modifications, the addition of nanoparticles, and inert pigments such as glass, iron oxide, and basalt particles [10,11,12,13,14]. Based on matrix material composites are classified into three types such as organic matrix composites, ceramic-based composites, and metal matrix composites [15,16].

Natural fiber hybrid composites became popular over synthetic fiber composites in recent years due to their lightweight, abundance, low cost, and high strength. These are mainly derived from plants, animals, and mineral resources. Sisal, palm, abaca, bamboo, hemp, flax, hemp, banana, and pineapple are some of the examples of natural (plant-based) fibers. Glass, silica, carbon, and ceramic are some of the examples of synthetic fibers. Synthetic fiber composites are not biodegradable, affect the environment by releasing harmful gases, and also cost-effective. Whereas natural fibers are readily available in nature and do not poses any harm to the environment [17,18,19]. The physical and mechanical properties of natural fibers and their composites are still being investigating.

In a broad sense, composite materials contain solid mass conveying elements is called reinforcement fill in delicate material is called matrix. Reinforcement affords rigidity and inflexibility, supporting the basic load. The matrix or else binder keeps up the location and introduction about reinforcement. Fundamentally, the content of the composite their personal, physical, and chemical properties are still well balanced they deliver a blend of characteristics that singular contents longing in fit for creating only [20,21]. The composite materials bear benefits over other regular materials because of their greater properties, for example, impact, elastic, and flexural strength, fatigue along stiffness properties, that empower the basic plan to be higher adaptable. Because of the benefits, they are generally eased on mechanical engineering applications like machine components, thermal control, internal combustion engines, electronic packaging, marine applications, construction industry, military applications, sports equipment, and the aerospace industry [22,23].

Chemical composition plays a significant role in plant-based natural fibers and varies from fiber to fiber. The fiber performance mainly depends on the aspect/ratio and cellulose content in the fiber materials. In plant-based fibers cellulose, hemicellulose, and lignin content play a prominent role in overall performance [24].

Reinforced hybrid materials are made by joining at least two distinct varieties of fibers in a distinctive matrix material. Hybridization of two sorts of filaments having particular lengths and widths offers a couple of central focuses over the use of both of the strands alone in a single polymer composite. By far most of the examinations are on the hybridization of natural fibers with glass strands to upgrade the properties [25]. Here the fibers act as the central load-carrying agents and the matrix material encompassing them in a position [26]. As reported by numerous scientists, the hybridization of different fibers under variable weight fractions tends to a gradual upsurge in mechanical, physical properties, and also cost-effective composite materials. The main benefit is mixing of the fibers can be done in various methods such as intermixed continuous fibers, intermixed discontinuous fibers, intermingled particulate fibers, aligned short fibers, and sandwich layers [27]. Sallam et al. [28] investigated the mode II fracture toughness of hybrid fiber reinforced concrete materials. The authors worked on the fracture toughness of four different hybridization patterns. The results showed that the fracture toughness improved in the hybrid composites. Though there are some advantages, it was sensitive to the hybridization patterns of fibers. The authors have given the suitable reasons for this behavior in a discussion part of the following paper concerning the readers’ doubts [29]. Sallam et al. [30] worked on the long-term behavior of normal-weight concrete contained hybrid nanoparticles subjected to gamma radiation. The experimental results showed that the addition of nanoparticles to the hybrid composites showed improvement in physical properties and mechanical properties.

The mechanical performance of epoxy resin-based hybrid composites with natural fibers such as hemp, flax, banana, pineapple, jute, sisal, bamboo, palm, abaca, and okra has been extensively studied by many researchers. Studies stated that natural fiber composites are conventional replacements for synthetic fiber composites. Alamri et al. [31] investigated the mechanical properties and water absorption behavior of recycled cellulose fiber reinforced epoxy composites. The results stated that the epoxy-based hybrid composites mechanical properties increased as fiber content increased. The effect of absorption on the mechanical properties was investigated by using SEM and FTIR analysis. The porosity and bonding between matrix material and reinforcement were observed. Sallam et al. [32] investigated the tribological and mechanical properties of epoxy reinforced by hybrid nanoparticles. Results showed that the addition of nanoparticles to the epoxy resin improved the wear resistance of epoxy and also there were some drawbacks such as reduction of tensile strength and modulus of elasticity. Colomer-Romero et al. [33] studied the comparison of mechanical properties of hemp-fiber bio composites fabricated with biobased and regular epoxy resins. The authors have stated that composites with normal epoxies exhibited improved results whereas bio-based epoxy resins caused fabrication difficulties due to their high viscosity nature and lead to a decrease in performance. Saba et al. [34] investigated the dynamic mechanical properties of oil palm nanofiller kenaf fiber epoxy hybrid composites. It has been found that the incorporation of nanofillers to the epoxy hybrid composites enhances its storage modulus and loss modulus. The authors concluded that the hybridization results in environmentally friendly nanocomposites, possessing superior damping properties and dynamic modulus. Jawaid et al. [35] studied the effect of oil palm and jute fiber treatment on the mechanical performance of epoxy hybrid composites. Results indicated that mechanical properties such as flexural and impact strength properties of altered fiber–reinforced hybrid composites enhanced as compared to unprocessed hybrid composites due to better fiber/matrix interfacial bonding, which was confirmed by scanning electron microscopy. Hanan et al. [36] investigated the mechanical performance of oil palm kenaf fiber-reinforced epoxy-based bilayer hybrid composites. Results showed that the hybridization of kenaf/palm fiber reinforced epoxy composites increased tensile and flexural properties. The inter bonding between matrix and filler material also improved and the results were observed by using SEM analysis. Though there was an improvement in the mechanical properties also poor bonding was observed in the scanning electron microscope analysis, this could be improved by using the chemical treatment of the fibers. Cordeiro et al. [37] studied the effect of composition and fiber modification on mechanical and dynamic properties of epoxy resin-based natural fiber peach palm composites. The authors concluded from the results that a very good mechanical response was observed for these composites even when 70 wt.% of fiber was used. Hanan et al. [38] worked on the characterization of hybrid oil palm empty fruit bunch woven kenaf fabric reinforced epoxy composites. In this research, they investigated the mechanical, physical, morphological properties of hybrid composites. Results obtained show that improvement in tensile and flexural properties whereas reduction in impact strength and is due to improper bonding or fabrication errors. The scanning electron microscopy (SEM) analysis results clearly show the various failure modes of the tensile fractured samples. The hybridization of palm fibers with the kenaf fibers has not given the proper results due to a lack of inter-bonding capabilities. Ramesh et al. [39] Studied the mechanical properties of glass/sisal/jute fiber hybrid composites. The results stated that glass/sisal fiber epoxy resin hybrid composites showed superior properties to jute/glass fiber composites. The addition of sisal to the glass fiber enhances the mechanical properties and also there are few drawbacks due to the presence of glass (synthetic) fibers such as biodegradability, moisture absorption properties. To overcome these problems an attempt has been made on Caryota and sisal natural fiber hybrid composites in the current work.

In this study, Caryota (C) and sisal (S) fiber-reinforced epoxy resin hybrid composites with varying weight fractions were fabricated by using the hand lay-up technique. The fibers were allowed for chemical treatment after the retting process. The fabricated samples were allowed for mechanical testing such as tensile properties, flexural properties, hardness, impact strength, and moisture properties. The scanning electron microscope was used to analyze the surface morphology, chemical composition, and porosity content in the composite samples. The mechanical performance of the fabricated samples was investigated and the results were compared.

## 2. Materials and Methods

### 2.1. Materials

Caryota and sisal fibers used as reinforcement materials and epoxy resin used as a matrix material. Sisal fibers are a type of leaf fiber extracted from the sisal plant. Caryota fibers are derived from fishtail palm trees. Both the fibers are readily available in nature. These plants are mainly cultivated in Asian countries since they always needed warm weather conditions. The chemical and mechanical properties of both sisal and Caryota fibers are given in Table 1. The thermoset polymer epoxy resin along with a hardener was used as a matrix material. The wood material was used for mold preparation for the fabrication process.

#### 2.1.1. Preparation of Fibers

**Figure 1:**
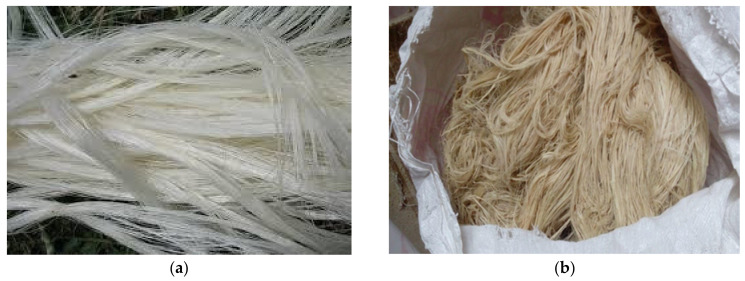
Sisal fibers (**a**) Caryota fibers (**b**) used for the fabrication of composites.

Fiber extraction is one of the foremost parts of the research. Caryota fibers were extracted from a fishtail palm tree by using a scraping machine. The scrapping machine is a combination of three rollers such as feed roller, serrate roller, and leaf scratching roller. Sisal fibers were extracted from the leaves of the agave sisalana plant. Once these fibers were extracted from resources, both were washed with still water and dried in sunlight. Then fibers were allowed for the chemical treatment process to increase the interface adhesion between fiber and matrix material. 2% HCl solution was prepared and each fiber was kept in the solution for 3–4 h separately and drawn out again washed in still water and dried at room temperature. Then the fiber laminates were separated by using mechanical combs by hand sitting patiently. After separating them, fibers were made into fine pieces using scissors according to the mold dimensions. The fibers used for fabrication purpose are shown in Figure 1.

#### 2.1.2. Weight Fraction of Materials

The weight fraction of reinforcements and matrix material used for the development of hybrid composites were considered based on the hybridization concept. It is defined as the fabrication of composites with two or more different fibers under the same resin matrix with a 0.4 weight fraction ratio (0.4 wf.). In this current research, a total of five different (varying weight fractions) composites were fabricated and the weight proportions are discussed in the following Table 2.

#### 2.1.3. Preparation of Matrix Material

Epoxy resin along with hardener was used as a matrix material for fabrication purpose. It was purchased from Sigma Aldrich. The weight proportions were considered as 10:1 ratio as per instructions. The required amount of epoxy and hardener were taken into a plastic container and stirred with a plastic stirrer for 3 to 4 min to get proper mix and left for 30 s. The chemical and mechanical properties of epoxy resin and hardener are given in Table 3.

#### 2.1.4. Fabrication of Composites

**Figure 2:**
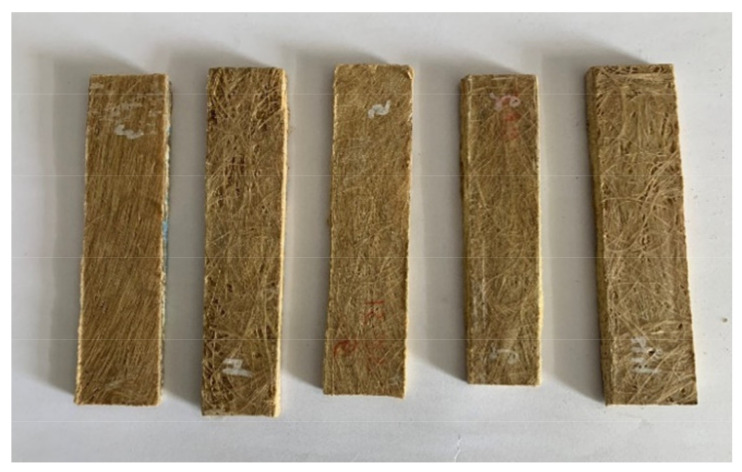
Sisal and Caryota fiber hybrid composites.

Caryota and Sisal fibers are measured for proportionate weight ratios as discussed above. The fibers were laid uniformly inside the mold before applying any resin to it. After arranging the fibers uniformly, they are compressed for a few minutes in the mold. The compressed fibers were laid over the coat of epoxy resin, ensuring uniform distribution of fibers. The epoxy resin mixture has been poured over the fibers uniformly and compressed for a curing time of 24 h, with a constant load of 5 kg. All samples are fabricated according to ASTM D 638M standards. The fabricated samples of Sisal/Caryota fiber hybrid composites are shown in Figure 2.

### 2.2. Methods

#### 2.2.1. Tensile Properties

**Figure 3:**
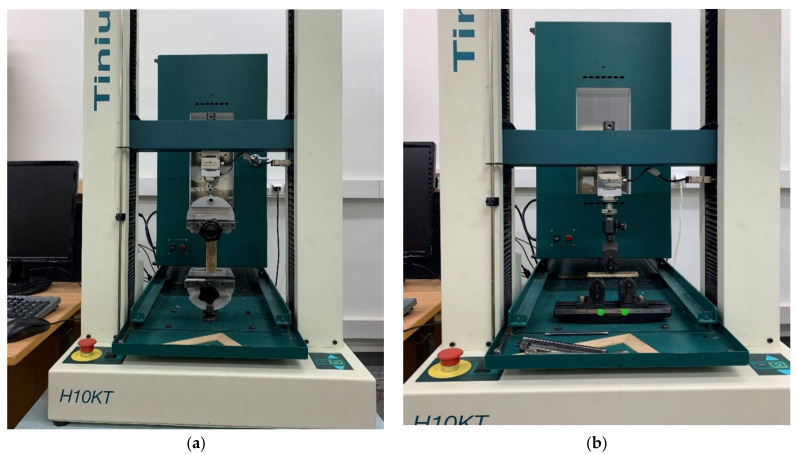
Tensile test (**a**) Flexural test (Three-point bending test) (**b**) on Tinus Olsen H10KT.

Tensile tests were performed to find out the in-plane tensile properties of polymer composites fabricated with matrix and reinforcements. In a broad sense, the tensile test is a measurement of the ability of a material to withstand external forces that tend to pull apart and to what extent the material stretches before breaking. The composite samples were tested on Tinus Olsen H10KT at a constant speed. A total of five samples were tested for each composite and the dimensions were considered as per ASTM D 3039 standards. The experimental setup used for the tensile test is shown in Figure 3 a. The tensile strength of composites was calculated by using the following relation.

(1) $$\sigma_{t}=\frac{P}{A}\;MPa$$

where $\sigma_{t}$ = tensile strength, *P* = maximum load, and *A* = cross sectional area.

The tensile modulus of a composite is the ratio of stress to elastic strain when the material is subjected to tension. The following relation was used to calculate the tensile modulus.

(2) $$E=\frac{FL}{Ae}\;MPa$$

where *E* = tensile modulus, *F* = maximum load, *A* = cross sectional area, and *e* = change in dimension.

#### 2.2.2. Flexural Properties

The flexural tests were performed to find out the maximum stress and strain when the composites are subjected to external loading. To find out the flexural strength, composite samples are allowed for 3-point bending tests on Tinus Olsen at a constant strain rate of 0.10 mm/min and a crosshead speed of 20 mm/min. The dimensions of samples were considered as per ASTM D 790 standards. The experimental setup used for the flexural test is shown in Figure 3b. The flexural strength was calculated by using the following relation.

(3) $$\sigma_{f}=\frac{3PL}{2bd^{2}}\;MPa$$

where $\sigma_{f}$ = flexural strength, *P* = maximum load, *L* = Span length, *b* = Width, and *d* = thickness.

Flexural modulus or tangent modulus is defined as the capability of a composite sample to deform. It is calculated from the tangent of the stress-strain curve. The following equation was used to calculate the flexural modulus.

(4) $$E_{B}=\frac{L^{3}m}{4bd^{3}}\;MPa$$

where $E_{B}$ = flexural modulus, *L* = span length, *m* = slope, *b* = width, and *d* = thickness.

#### 2.2.3. Impact Strength Test

**Figure 4:**
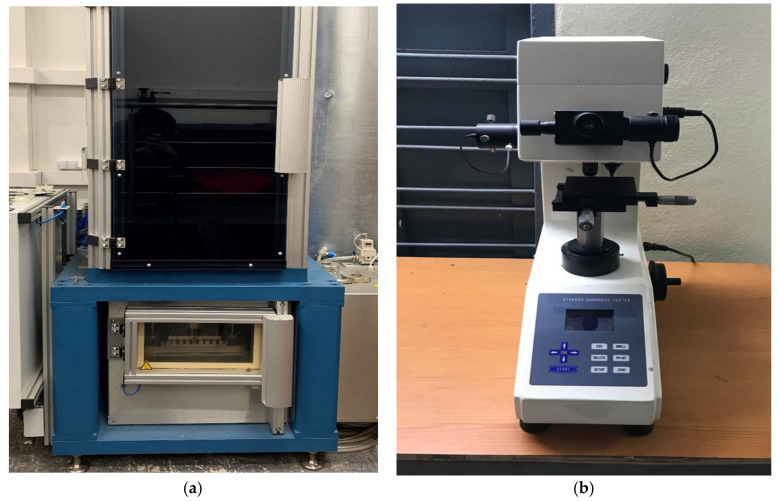
Coesfeld Magnus Impact testing system (**a**) Micro Vickers hardness tester (**b**).

This test is used to define the ability of composite material to withstand the sudden shock loads. This test was carried on a Coesfeld Magnus impact testing machine. The machine consists of a high-speed drop tower with a maximum impactor speed of 40 m/s. The drop tower swung from a set height and which on releasing possess fixed kinetic energy. The test samples were placed like a simply supported beam on the resting position of the impact machine. The tests were conducted as per ASTM D 6110 standards. The experimental setup used for this test is shown in Figure 4a.

#### 2.2.4. Vickers Hardness Test

This test is used to find the ability of composite material to resist indentation, scratch, or surface abrasion. In this work, the hardness test was carried out on the Micro Vickers hardness testing machine of model DH85 and Daksh made. It has a load range of 0.2–5 kgs and a measuring range of 5–3000 HV. The measuring magnification and observer magnification are given as 500X and 100X respectively. The experimental setup used for this test is shown in Figure 4b.

#### 2.2.5. Moisture Absorption Test

A Moisture absorption test is used to find out the amount of water absorbed by a composite material when it is exposed to moisture. To find out the rate of absorption, composite samples were placed in a beaker full of distilled water. The sample dimensions were considered as per ASTM D 570 standards. The readings are noted for continuous-time intervals for one week. A total of five samples were tested for each composite material. The rate of absorption was calculated by using the following relation.

(5) $$\%\;Weight\;gain=\;\frac{W_{2}-W_{1}}{W_{1}}\;\times 100$$

where *W*_2_ = Final weight, *W*_1_ = Initial weight.

#### 2.2.6. Scanning Electron Microscopic Analysis.

The failure studies, surface morphology, and chemical composition of the composite samples were evaluated by using the scanning electron microscope (S-3400N from Hitachi) with an energy dispersive X-ray spectrometer (Quad 5040 from Bruker, Billercia, MA, USA).

## 3. Results

### 3.1. Tensile Properties

The analytical results for Caryota and sisal fiber hybrid composites obtained from tensile tests are mentioned in the following Table 4. All the composites specimens were tested as per the standards and for each composite, a total of five samples were tested to note down the average values. The tensile stress was applied in the longitudinal to the fibers direction and speed was maintained as 2mm/min. The tests were conducted at 21 °C temperature and relative humidity as 28%. The weight fractions of composites are 40C/0S, 25C/15S, 20C/20S, 15C/25S, 0C/40S.

**Figure 5:**
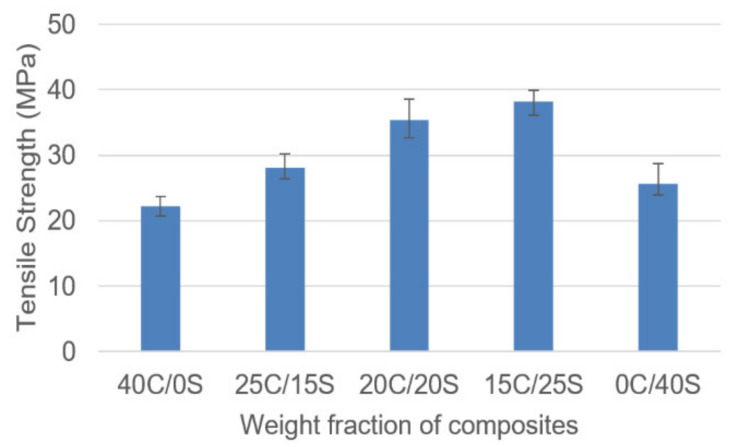
Tensile strength vs. weight fraction of C/S composites.

Figure 5 shows the tensile strength behavior for Caryota and sisal fiber hybrid composites with varying weight fractions. From the results, the tensile strength values of 15C/25S hybrid composites are inferior as compared to the other composites. It showed the tensile strength as 38.2 MPa whereas 20C/20S showed 35.4 MPa. The single fiber composites showed the least tensile strength as 22.2 and 25.7 MPa. The hybridization effect is noticed in this case. It was observed that the tensile strength values among the hybrid composites increased with an increase in sisal fiber content in it. The reason for the above can be attributed in two ways. An increase in sisal fiber content increases the strength of the composite. However, higher individual fiber content leads to the agglomeration of fibers, hence loss of strength. Poor bonding between matrix and reinforcement material at the interface might be another reason for lower the tensile strength value.

**Figure 6:**
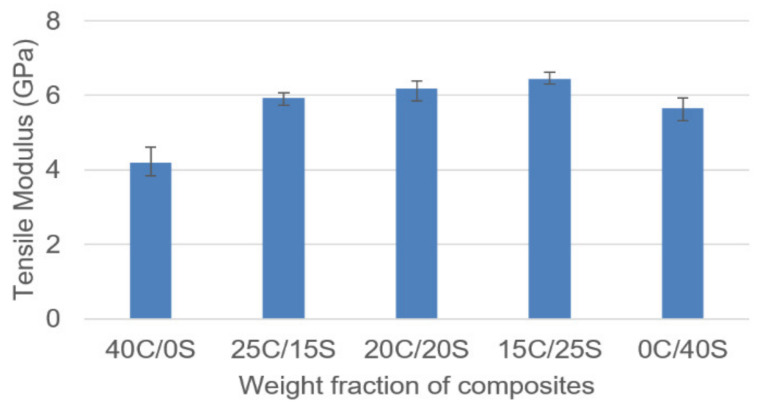
Tensile modulus vs. weight fraction of C/S composites.

The tensile modulus of various weight fractions of composites is given in the following Figure 6. Among all the composites hybrid composites showed superior properties over single fiber composites and the values are close to each other. Single fiber Caryota composites showed the least tensile modulus among all the composites and 15C/25S composites showed the highest tensile modulus.

### 3.2. Flexural Properties

The data presented in Table 5 demonstrates that the flexural properties for all the composites. Flexural tests were performed by using three-point bending tests and the dimensions of the test samples are taken from ASTM standards. The flexural test results include displacement and strength. The tests were conducted at 21 °C temperature and relative humidity as 28%. For each composite, a total of five samples were tested and the values are noted.

**Figure 7:**
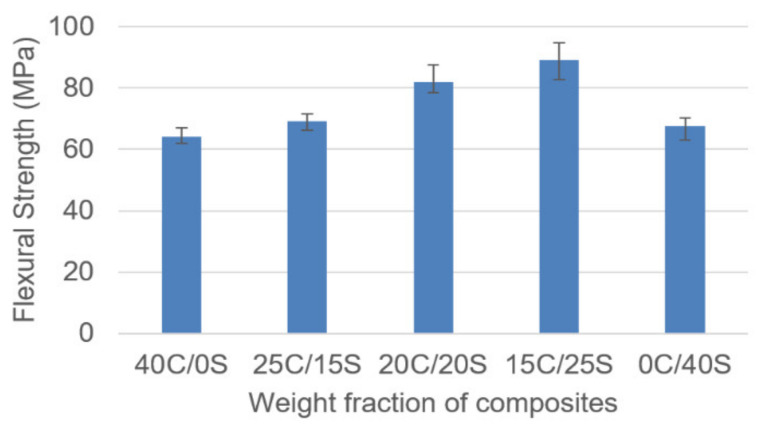
Flexural strength vs. weight fraction of C/S composites.

Figure 7 shows the relation between flexural strength and weight fraction of the fabricated composites. As it can be seen from the below figure that the hybrid composites showed superior flexural properties than single fiber composites. 15C/25S hybrid composites showed the highest flexural strength as 89.16 MPa and single fiber Caryota composites showed the least strength as 64.09 MPa. Hybrid composites exhibited good flexural properties which indicate that the materials have brittle properties. It was observed that the flexural properties trend is similar to the tensile properties. The loss in strength of single fiber composites can be explained as (1) early failure of the composite upon external loading. (2) weak interfacial bonds between fiber and matrix material leads to the clustering of fibers.

**Figure 8:**
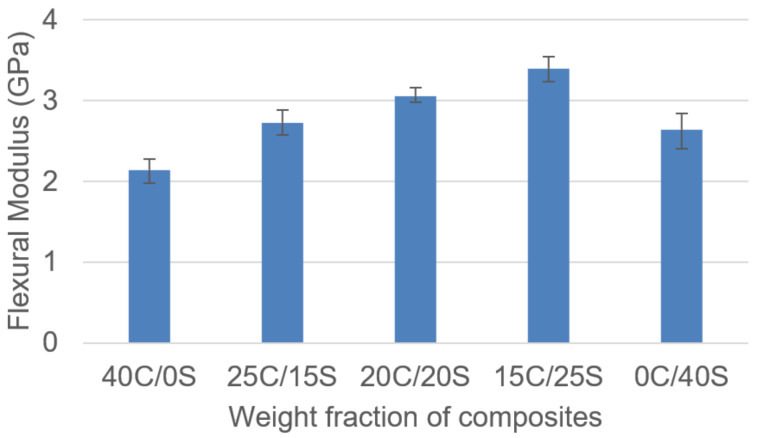
Flexural modulus vs. weight fraction of C/S composites.

The results presented in Figure 8 show the evolution of flexural modulus according to the fiber weight fractions. It was observed from the results that single fiber Caryota and sisal fiber composites showed a lower flexural modulus than Caryota sisal hybrid composites. Single fiber composites showed 2.14 GPa and 2.64 GPa respectively.

The flexural strength is higher than the tensile strength. Indeed, the dimensions of the test samples are similar for both tests. Only at the center of the composite is stressed in the three-point bending test while the whole is in tension test, then fewer defects are involved in the 3-point bending test. Whereas tangent modulus or tensile modulus is more than the flexural modulus. The reason can be attributed to, in a tensile test, the maximum tensile stresses are experienced throughout the entire volume (and surface area) of composites whereas, in bending test, the maximum tensile stresses are conversely concentrated in a small region on the top surface.

### 3.3. Impact Test

**Figure 9:**
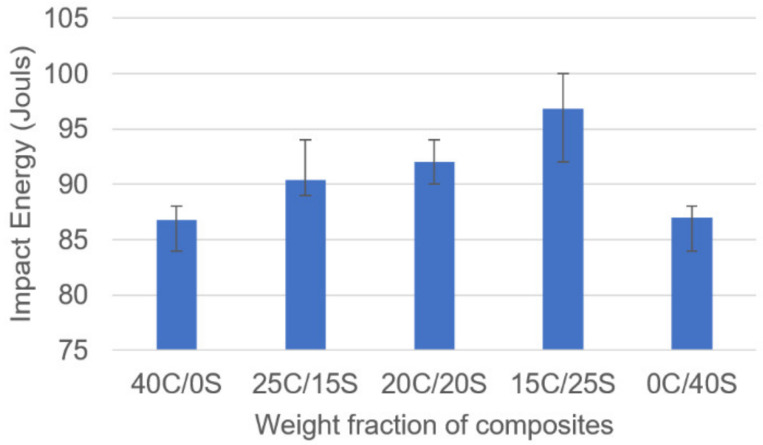
Impact strength vs. fiber weight fractions.

The data presented in Figure 9 was obtained from the impact tests. The specifications of the samples were taken from ASTM standards and the tests were conducted at 21 °C temperature and relative humidity as 28%. It is well known that the impact strength of the composites mainly depends on the properties of reinforcement and matrix material presented in it. It was observed that the hybrid composites showed superior impact strength over single fiber composites. Single sisal fiber composites exhibited the least impact strength as 83.5 Joules.

### 3.4. Vickers Hardness Test

**Figure 10:**
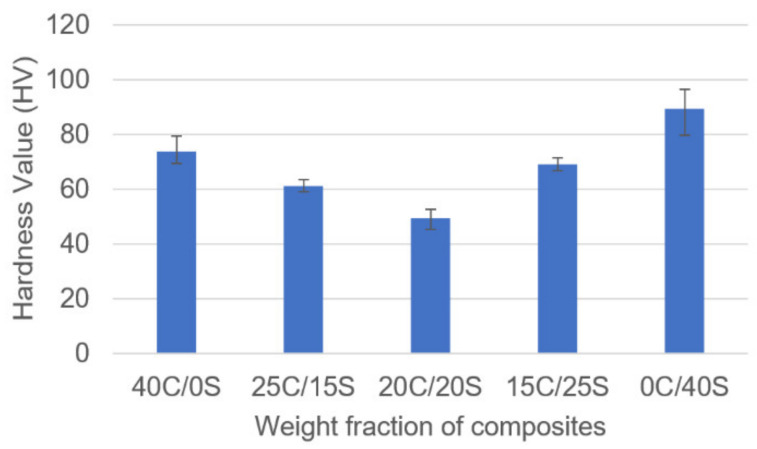
Hardness vs. weight fraction of C/S composites.

The hardness values of various weight fractions of composites are given in Figure 10. It was observed from the results, single fiber composites showed the highest hardness values whereas hybrid composites showed the least hardness values. The hybridization impact is not noticed in this case. 20C/20S composite showed the least Vickers hardness values among all composites.

### 3.5. Moisture Analysis

The amount of moisture absorbed by the composites was calculated by using the moisture test. The samples were allowed for the heat treatment process (placed in an oven at 60 °C for 15 min time to eliminate the moisture in it) before placing them in the water container. The sample dimensions were taken from the ASTM standards and the test was conducted at 21 °C temperature and relative humidity as 28%.

**Figure 11:**
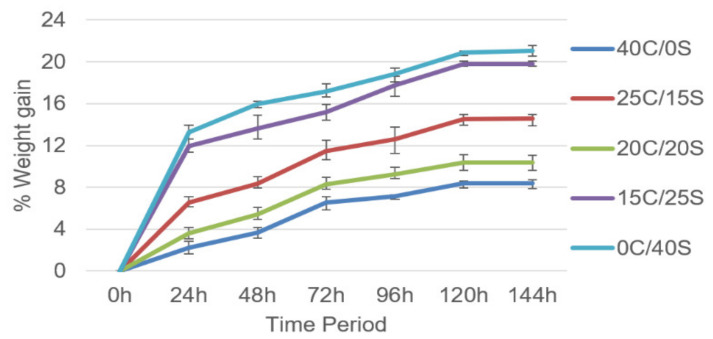
Weight gain in % by the composites vs. time period.

Figure 11 shows the % weight gain by the composites with varying weight fractions to time. It was observed that all the composites samples responded to the moisture (water) when it is exposed. The amount of weight gain by the composites increased with time up to 120 h and remains constant. All hybrid composites showed intermediate results whereas single fiber composites showed the least and highest percentage of weight gain. The reason can be attributed to the presence of void content (porosity) in the composites and the chemical composition (cellulose content) of the fibers. Because the oxygen ($C_{6}H_{10}O_{5}$) in the cellulose reacts to the hydrogen in the moisture and forms the hydrogen-oxygen bonds with it. Sisal fibers contain more cellulose percentage than Caryota, hence it observed more % of water. The porosity in the composites specimens was observed by using SEM analysis and the results were mentioned in Figure 17. It was observed that the hybrid composites have an intermediate percentage of void places compared to the single fiber composites. The presence of void places attracts the water molecules hence the percentage gain is more.

### 3.6. Scanning Electron Microscopic (SEM) Analysis

The SEM images of the flexural, tensile tests fractured composites and chemical composition at the surface and fractured specimens were taken to analyze the presence of voids, cracks, and the reinforcement and matrix adhesion (bonding) behavior.

**Figure 12:**
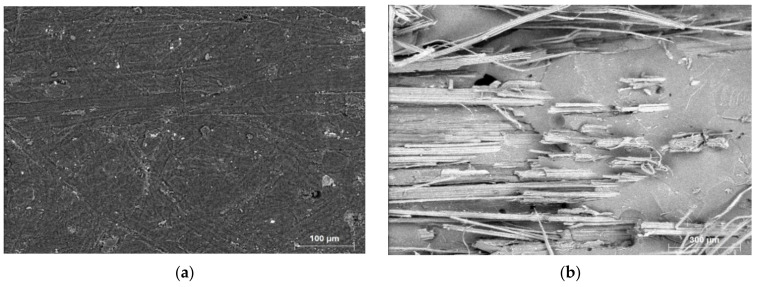
SEM micrographs of single sisal fiber composite (0C/40S) (**a**) surface and (**b**) fractured position.

The above Figure 12 shows the micrographic spectrum acquisition for single sisal fiber composite to analyze the chemical composition in it.

**Figure 13:**
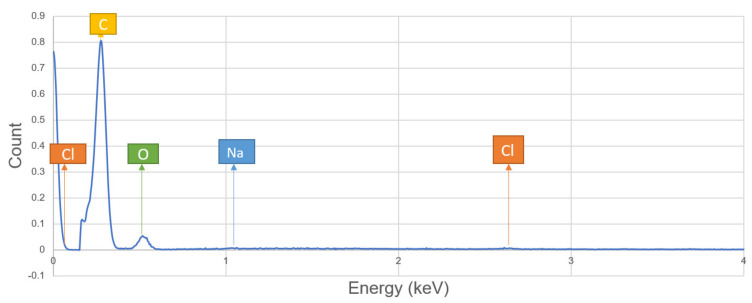
Spectrum analysis of the chemical structure of single sisal fiber hybrid composite surface position obtained at an intensity of 4 keV.

Based on the spectrum analysis of a specimen, single sisal fiber hybrid composites the presence of composition on the surface is shown in Figure 13. The data presented in the above graph is shown in the following Table 6. The structure is expressed by the atomic weight and number of each element presented in it. The weight % of carbon is more than the weight % of oxygen, this reflects that the matrix concentration is more at the surface of the composite than reinforcement. Due to this smooth surface finish will occur to the composite.

**Figure 14:**
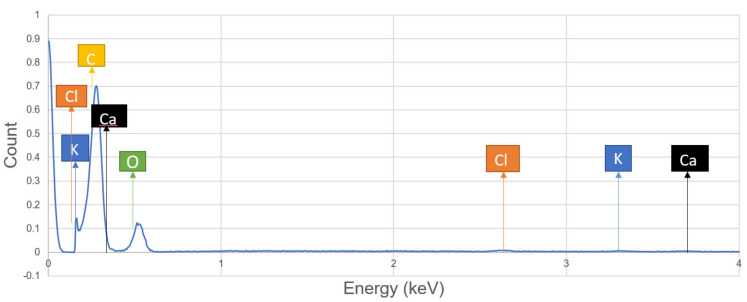
Spectrum analysis of the chemical structure of single sisal fiber hybrid composite fractured position obtained at an intensity of 4 keV.

Based on the spectrum analysis of a specimen, single sisal fiber hybrid composites the presence of composition on the surface is shown in Figure 14. The data presented in the above graph is shown in the following Table 7. The structure is expressed by the atomic weight and number of each element presented in it. It was observed that the weight % of carbon is nearly equal to the weight % of oxygen, this reflects that the matrix concentration and reinforcements are in proper proportion inside the composite. This reflects strong bonding between matrix and reinforcement in the composite and also, improvement in the strength of the composite.

After the analysis of chemical concentration presented at the surface and fractured position of single sisal fiber hybrid composite, we can conclude that the percentage of carbon is increased in the surface compared to the fractured (inside) position which denotes that epoxy concentration is more at the surface. Whereas oxygen percentage more at the fractured position which denotes that fiber concentration is more at the breakpoint.

**Figure 15:**
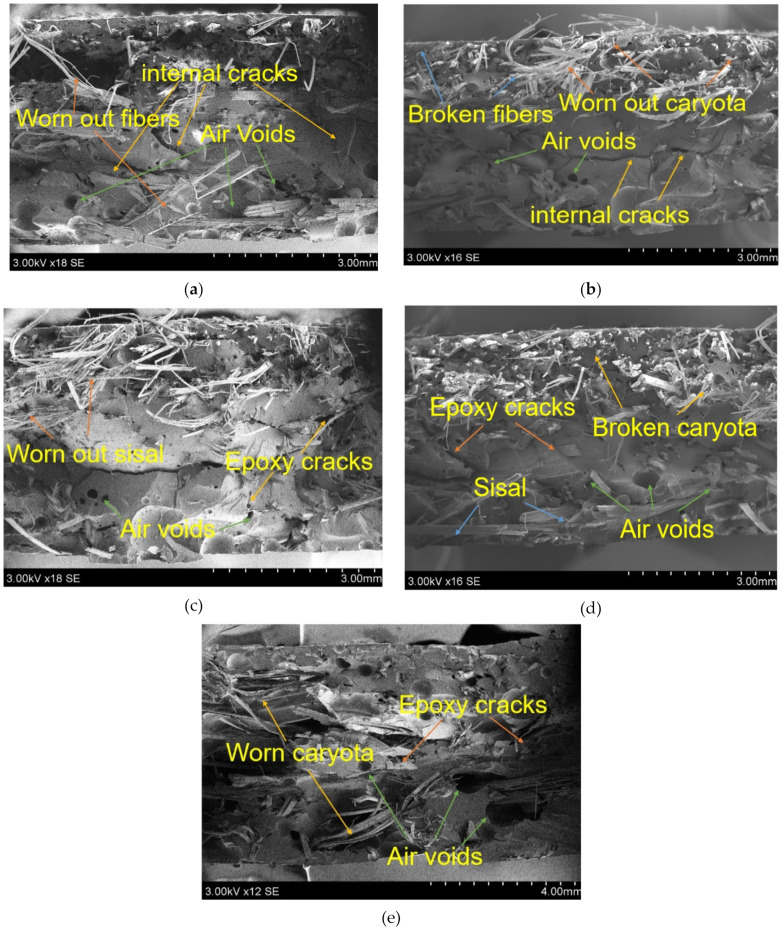
SEM images for fractured composites of flexure test specimens (**a**) 0C/40S, (**b**) 15C/25S, (**c**) 20C/20S, (**d**) 25C/15S, (**e**) 40C/0S.

Figure 15 shows the fracture surface of the flexural test composites as a function of hybridization. A brittle fracture of reinforcement and matrix material was observed from the results. The reason can be attributed to the strong interfacial bonds between fibers and epoxy resin. It can be seen from the above figures that the presence of voids and cracks leads to weakening the strength of the composites. It was observed that single fiber composites (Figure 15a,e)) have more cracks and air gaps in them when compared to the hybrid composites, this is due to lack of fiber/resin adhesion and poor bonding nature.

**Figure 16:**
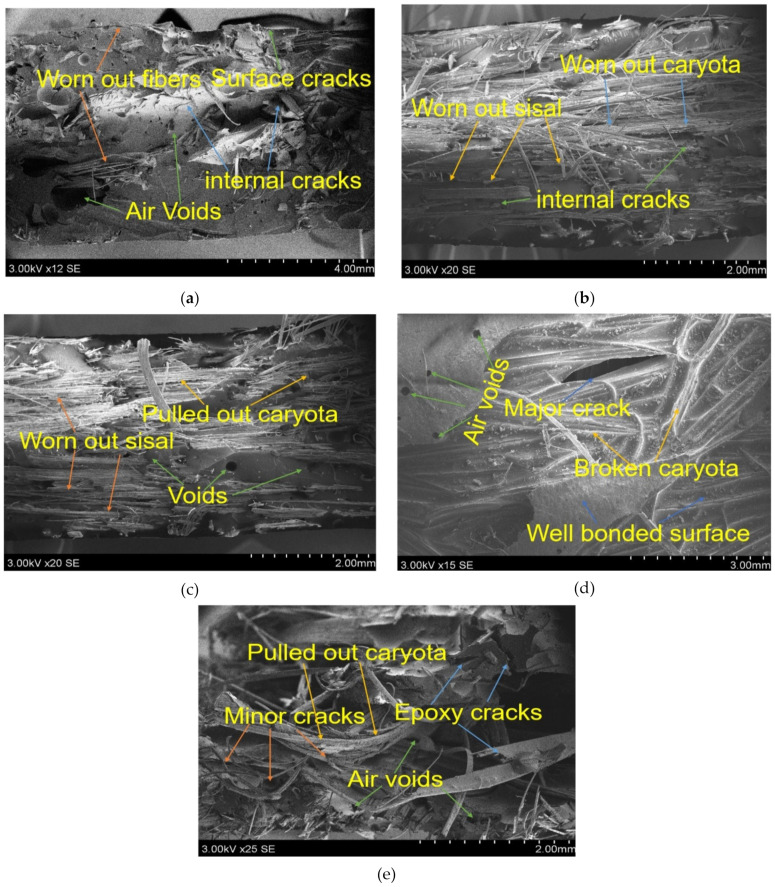
SEM images for fractured composites of tensile test specimens (**a**) 0C/40S, (**b**) 15C/25S, (**c**) 20C/20S, (**d**) 25C/15S, (**e**) 40C/0S.

The fracture properties of composites after the tensile test were inspected through SEM and it is given in Figure 16. it is observed that the cluster of matrix shards cling to the fiber reinforcements and enclosed it. This helps the composite to worn out when the external load acting on it when it reaches its maximum strength. The fiber pulled out needs extra strength compared to fiber fracture. it can be seen that in the case of single fiber composites (Figure 16a,e) the fibers are separated from the matrix due to poor adhesion. Wherein the case of hybrid composites some of the matrix material is presented at the top of fibers. This indicates the strong crosslinking reaction between reinforcement and matrix materials. That gives extra support to the composites to worn out. Moreover, it was observed that the presence of cracks and void places are less in hybrid composites compared to the single fiber composites.

**Figure 17:**
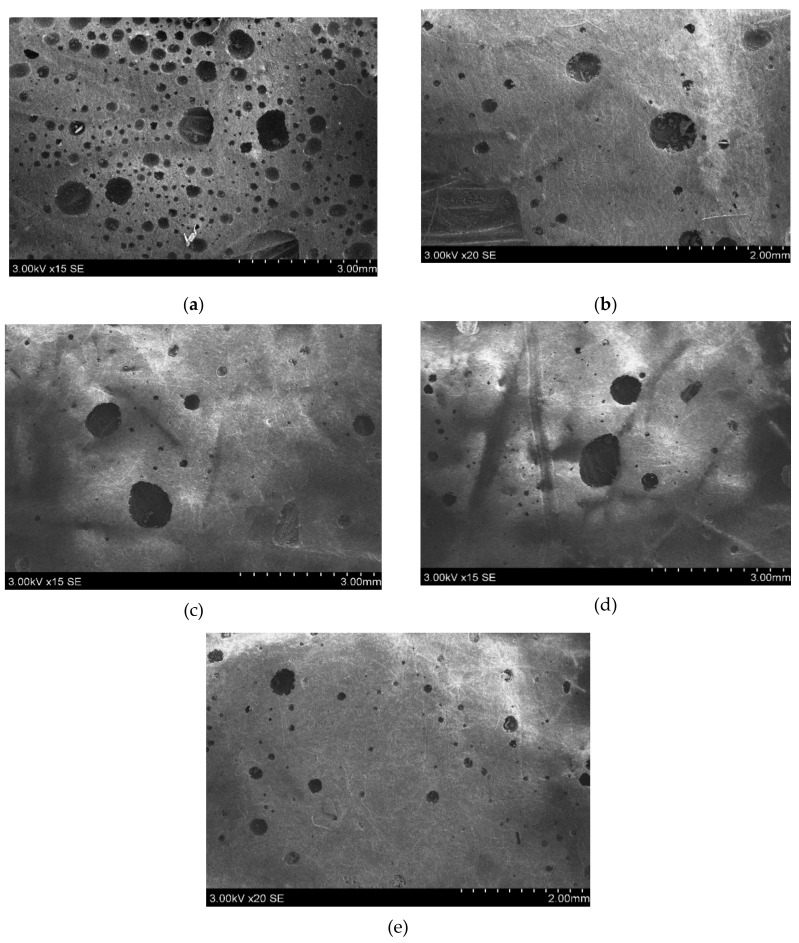
SEM images for porosity (void content) presented in the fabricated composites (**a**) 0C/40S, (**b**) 15C/25S, (**c**) 20C/20S, (**d**) 25C/15S, (**e**) 40C/0S.

The presence of air voids and gaps in the composite surfaces may lead to weakening the strength of the composite and also the composite may absorb more water when it faces the wet medium. Figure 17 shows the presence of porosity content in all the fabricated composites, it was observed that 0C/40S composite has the more void percentage whereas 40C/0S composite has the least percentage of void content. All the hybrid composites have intermediate percentages and close to each other. The reasons can attribute as (1) strong internal bonding between reinforcement and matrix in the hybrid composites. (2) fabrication errors. (3) atmospheric conditions such as temperature and humidity while fabricating the composites.

## 4. Discussion

Previous research was concentrated on the fabrication of banana, pineapple, hemp, flax, and sisal fibers-based hybrid composites for lightweight and biomedical applications [43,44,45,46,47,48,49]. The present research was concentrated on developing the hybrid composites with Caryota (derived from fishtail palm plants) and sisal fiber-based hybrid composites with varying weight fractions.

The Caryota and sisal fiber-reinforced epoxy resin-based hybrid composites were fabricated by using the hand layup technique. The developed hybrid composites have shown superior mechanical properties. In the previous research, banana/pineapple-based hybrid composites showed flexural properties ranging from 37 to 53MPa and hemp/flax hybrid composites showed that in between 45 to 63 MPa whereas Caryota and sisal-based hybrid composites showed in between 65 to 89 MPa which is far higher than other hybrid composites. Similarly, there is a considerable improvement in the tensile properties as well.

For future aspects, implementation of nanoparticles such as nano clay. ferrous oxide and silver particles in bio-based epoxy resin foreseen. Moreover, the orientation of fiber materials in the composite materials has to be considered because, previous researchers proved that, the orientation of reinforcement may increase the torsional strength and also crosslinking between matrix and fiber material. The bio-based composites could be used for biomedical and microfluidic applications.

## 5. Conclusions

Caryota and sisal fiber-based epoxy resin hybrid composites were developed by using the hand layup technique and the mechanical properties were investigated. Experimental results stated that hybrid composites showed superior properties. From the tensile test, it was found that 15C/25S hybrid composites have shown 38 MPa whereas single fiber composites (0C/40S, 40C/0S) showed 25 and 22 MPa respectively. From flexural properties, it was found that the hybrid composites showed improved flexural properties than single fiber composites. The 15C/25S hybrid composites showed flexural strength and flexural modulus as 89.16 MPa and 3.40 GPa. Single Caryota fiber composites showed the least strength and modulus as 64.09 MPa and 2.14 GPa. Coming to the impact strength 15C/25S composites showed as 97 joules and in single sisal fiber composites, it was observed that 83.5 joules. in all the tests hybridization effect was observed clearly except the hardness test. In the hardness test, single fiber composites showed superior properties to hybrid composites. The reason could be fabrication errors or poor bonding between matrix and reinforcement material.

Thus, SEM analysis showed that hybrid composites have fewer defects such as voids, minor and major cracks, poor adhesion between matrix and reinforcement material than single fiber composites.

Moisture absorption measurements stated that all the composites showed a constant increase up to 120 h and remain constant. Hybrid composites showed intermediate results whereas single fiber composites showed the highest and lowest rate of absorption. By considering all the results Caryota and sisal fiber hybrid composites showed superior mechanical properties than single fiber composites. And Caryota and sisal fibers are potential alternatives in fiber composite fabrication.

## Figures and Tables

**Table 1:** The chemical composition and mechanical properties of natural fibers [40,41].

Property	Sisal Fibers	Caryota Fibers
Cellulose (%)	50–78	37–47
Hemicellulose (%)	10–14	25–34
Lignin (%)	8–11	18–23
Density ($g/cm^{3}$)	1.45	0.7–1.55
Young’s modulus (GPa)	9.4–22	1–9
Elongation at break (%)	3–7	2–4.5
Moisture (%)	11	13–15
Microfibrillar angle (°)	20–25	14–18

**Table 2:** Fiber weight fractions.

Composite Type	Caryota Fiber (%)	Sisal Fiber (%)	Total Fiber Volume (%)	Total Resin Volume (%)
40C/0S	40	0	40	60
25C/15S	25	15	40	60
20C/20S	20	20	40	60
15C/25S	15	25	40	60
0C/40S	0	40	40	60

**Table 3:** The chemical and mechanical properties of Epoxy and Hardener [42].

Property	Epoxy Resin	Hardener
Type	Araldite LY 556	Ardur HY 951
Mixing proportion	10	1
Color	Pale	Brown
Specific gravity	1.14	1.02
Density ($g/{\mathrm{cm}}^{3}$)	1.15–1.18	0.97–0.99
Viscosity at 25 °C (MPa)	550	600
Curing time (h) at 23 °C	24–32	24–32
Pot life (min) at 23 °C	35	35

**Table 4:** Tensile strength properties.

Composites	Tensile Strength (MPa)	Tensile Modulus (GPa)
40C/0S	22.2	4.20
25C/15S	28.1	5.92
20C/20S	35.4	6.19
15C/25S	38.2	6.44
0C/40S	25.7	5.66

**Table 5:** Flexural strength properties.

Composites	Break Load (N)	Flexural Strength (MPa)	Flexural Modulus (GPa)
40C/0S	267	64.09	2.14
25C/15S	287.7	69.05	2.73
20C/20S	341.2	81.89	3.06
15C/25S	371.5	89.16	3.40
0C/40S	282.1	67.70	2.64

**Table 6:** Chemical structure of single sisal fiber hybrid composite at surface position.

Element Number	Element Symbol	Element Name	Series Name	Atomic Weight	Weight Conc. (%)	Atomic Conc. (%)
6	C	Carbon	K-series	12	68.68	74.66
8	O	Oxygen	K-series	16	30.75	25.09
11	Na	Sodium	K-series	23	0.17	0.10
17	Cl	Chlorine	K-series	35.5	0.40	0.15

**Table 7:** Chemical structure of single sisal fiber hybrid composite at fractured position.

Element Number	Element Symbol	Element Name	Series Name	Atomic Weight	Weight Conc. (%)	Atomic Conc. (%)
6	C	Carbon	K-series	12	52.56	59.98
8	O	Oxygen	K-series	16	46.18	39.57
17	Cl	Chlorine	K-series	35.5	0.46	0.18
19	K	Potassium	K-series	39.09	0.38	0.13
20	Ca	Calcium	K-series	40.08	0.42	0.14

## Data Availability

Data sharing is not applicable.
